# Electrochemical Coagulant Generation via Aluminum-Based Electrocoagulation for Sustainable Greywater Treatment and Reuse: Optimization Through Response Surface Methodology and Kinetic Modelling

**DOI:** 10.3390/molecules30183779

**Published:** 2025-09-17

**Authors:** Benan Yazıcı Karabulut

**Affiliations:** Department of Environmental Engineering, Harran University, 63050 Sanliurfa, Türkiye; benanyazici@harran.edu.tr

**Keywords:** electrocoagulation, aluminum electrodes, chemical oxygen demand removal, sustainable wastewater treatment, sludge characterization, response surface methodology

## Abstract

This study investigates the operational performance and optimization of a real greywater treatment system utilizing aluminum (Al)-based electrocoagulation (EC). The EC process was systematically evaluated and optimized through Response Surface Methodology (RSM) using the Box–Behnken Design (BBD), focusing on three critical parameters: pH, current density, and electrolysis time. Greywater samples collected from domestic sources were characterized by key physicochemical parameters including pH, COD, TSS, turbidity-ty, and electrical conductivity. The electrochemical treatment was conducted using a batch reactor equipped with Al electrodes in a monopolar configuration. Scanning Electron Microscopy (SEM), Energy Dispersive X-ray Spectroscopy (EDS), X-ray Diffraction (XRD), and Fourier-Transform Infrared Spectroscopy (FTIR) were employed to characterize both the electrodes and the generated sludge. Results revealed a maximum COD removal efficiency of 86.34% under optimized conditions, with current density being the most influential factor, followed by its significant interaction with pH. The developed quadratic model exhibited high predictive accuracy (R^2^ = 0.96) and revealed significant nonlinear and interaction effects among the parameters. Sludge characterization confirmed the presence of amorphous aluminum hydroxide and oxyhydroxide phases, indicating effective coagulant generation and pollutant capture. The treated greywater met physicochemical criteria for non-potable reuse, such as agricultural irrigation, supporting resource recovery objectives. These findings demonstrate that EC is a low-waste, chemically efficient, and scalable process for decentralized wastewater treatment, aligning with the goals of sustainable chemical engineering.

## 1. Introduction

Greywater is the wastewater that comes from non-toilet activities in the home, like bathing, doing laundry, and washing dishes. It makes up about 50–80% of all household wastewater [1,2]. Greywater usually has fewer organic pollutants and pathogens than blackwater, which makes it a good choice for on-site treatment and reuse, especially in places where water is scarce [3]. The secure and effective reutilization of greywater can markedly reduce the strain on freshwater resources and advance sustainable water management and environmental conservation objectives [3,4].

Traditional greywater treatment techniques—such as physical filtration, activated sludge processes (a common form of biological treatment), and chemical coagulation—often face several practical and operational limitations that hamper their reuse and sustainability potential. Filtration systems generally necessitate a substantial land area, regular backwashing, and constant maintenance to prevent clogging [5]. Activated sludge processes require highly skilled workers, constant aeration, and energy-intensive operations that may not be cost-effective in decentralized or resource-limited settings [6]. Chemical coagulation, on the other hand, involves the constant dosing of chemical coagulants—such as alum or ferric salts—with the attendant handling and disposal of sludge, thus complicating operation and chemicals reliance [7]. Collectively, these drawbacks—in terms of significant space requirements, large chemical inputs, energy demands, and the need for round-the-clock expert management—make them less suitable for small-scale or onsite greywater recycling programs. Consequently, interest in more compact, low-chemical, and energy-efficient alternatives like electrocoagulation (EC) is on the rise. EC is a promising technique, given its simplicity and enhanced efficiency in terms of pollutant removal. The method employs metallic electrodes, typically composed of aluminum or iron, to generate coagulant species through the process of electrochemical dissolution. The elimination of impurities is facilitated through a combination of charge neutralization, sweep flocculation, and adsorption. Aluminum has attracted significant attention as a potential electrode material due to its mechanical strength, corrosion resistance, and potential efficacy under prolonged or semi-continuous treatment conditions [8,9]. The minimal maintenance requirements and reusability of aluminum render it economically attractive for practical applications [10,11].

It is imperative to acknowledge the pivotal role that numerous operating parameters play in the assessment of EC system performance. These parameters encompass initial pH, current density, voltage, electrode distance, and electrolysis time. The optimal pH range for greywater treatment is predominantly within the range of 6.0 to 8.0, a condition that fosters the generation and stability of flocs for the effective removal of pollutants such as turbidity and chemical oxygen demand (COD) [3,4]. However, due to the non-linear interactions among these variables, systematic optimization is required to achieve maximum performance.

Response Surface Methodology (RSM) is widely used in environmental studies to better understand how different operational parameters work together and influence treatment performance. It is evident that one of the most practical designs within RSM is the Box-Behnken Design (BBD), which has been shown to strike an optimal balance between experimental cost and accuracy. In contrast to full factorial designs, BBD necessitates a reduced number of experiments and circumvents the necessity for extreme conditions, rendering it especially well-suited for delicate systems such as EC [12]. The application of BBD facilitates the exploration of how multiple factors interact, the identification of optimal treatment conditions, and the acquisition of profound insights into the overall behavior of the process. This approach has been demonstrated to enhance the efficiency of EC systems and to support their potential application in real-world water treatment scenarios [4,9]. The most recent studies have investigated the combination of EC with other technologies, including biological treatment, filtration, and membrane separation, in hybrid treatment systems. The aim of these integrated systems is to improve treatment efficiency, enable nutrient recovery, and ensure long-term sustainability in wastewater treatment applications [13,14].

This study will examine the effectiveness of an Al-based EC unit in the treatment of greywater. The main focus will be the utilization of Response Surface Methodology through Box-Behnken Design in determining optimum operating parameters. The results seek to provide practical, affordable, and sustainable means of reusing greywater in both urban and rural settlements.

## 2. Results and Discussion

### 2.1. Greywater Characteristics

The initial physicochemical characteristics of the greywater samples are listed in Table 1. The physicochemical properties of untreated greywater samples are summarized in Table 1. These initial values provide insights into the contamination level and treatment needs of domestic greywater prior to processing. The pH value of the greywater sample was recorded at 8.1, indicating a slightly alkaline condition. This is consistent with observations in various studies that reported pH values in greywater typically range between 6.5 and 9 due to the use of alkaline cleaning agents and soaps in households [15,16]. The pH value is central to determining the effectiveness of both biological and chemical treatment methods. For instance, the effectiveness of coagulation and microbial activity is significantly influenced by pH variations [17]. The Chemical Oxygen Demand (COD) was found to be 624 mg/L, representing a significant organic load. The measurement is consistent with the findings of previous studies, which have indicated that greywater from kitchen and laundry sources can exhibit a COD ranging from 200 to 1000 mg/L, contingent on usage patterns [16,18]. A high COD reading means that there are a lot of biodegradable and non-biodegradable organic compounds, like oils, fats, surfactants, and food waste. These high levels of organic matter show that both primary and secondary treatment steps are needed. The raw greywater exhibited an electrical conductivity (EC) value of 1350 µS/cm, which lies within the typical range of 500–2500 µS/cm reported for domestic greywater in the literature [6,19]. Following EC under optimized conditions, the conductivity decreased to 865 µS/cm. This reduction can be explained by the formation of coagulant hydroxides that facilitate the removal of dissolved ions through charge neutralization, sweep flocculation, and subsequent precipitation. Specifically, the partial elimination of sodium and sulfate ions derived from detergents, as well as calcium/magnesium complexes and other inorganic salts, contributed to the overall decline in conductivity. Although international standards such as World Health Organization (WHO) [20], United States Environmental Protection Agency (USEPA) [21], and the European Union Regulation 2020/741 [22] do not specify threshold values for EC in reclaimed water, salinity is a critical parameter for agricultural reuse. According to the Food and Agricultural Organization (FAO) irrigation water quality guidelines, waters with EC < 750 µS/cm pose a low salinity hazard, while 750–2250 µS/cm indicates a moderate hazard [23]. In this context, the raw greywater presented a moderate salinity risk, whereas the treated greywater approached the threshold between low and moderate hazard. This improvement enhances its suitability for irrigation and other non-potable reuse applications. Such elevated values are generally attributed to the presence of dissolved ions originating from detergents, soaps, food residues, and cleaning agents. It is imperative to acknowledge the significance of EC levels in the selection of treatment technologies, particularly in contexts where the objective is to reuse water for irrigation. Elevated salinity has been observed to exert a detrimental influence on soil permeability and the well-being of plants [24,25]. Turbidity was measured at 74 NTU, revealing a high level of suspended particles and colloidal matter in the sample. This result is aligned with those reported in the literature, where greywater turbidity is often in the range of 50 to 200 NTU. High turbidity can affect the transmission of light during disinfection treatments like ultraviolet (UV) treatment, which may reduce the effectiveness of such treatments. Furthermore, the concentration of total suspended solids (TSS) was measured at 286 mg/L, representing the presence of a considerable amount of particulate material, such as textile fibers, hair, skin cells, and food waste. This result is comparable to data reported in several studies, where TSS concentrations are typically in the range of 100 to 400 mg/L [15,26]. TSS is a key parameter for assessing whether pre-filtration or sedimentation steps are necessary. It has been demonstrated that technologies such as sand filtration are highly effective in significantly reducing TSS (total suspended solids) and turbidity values [27]. These findings corroborate earlier research highlighting the complex and heterogeneous nature of greywater, whose composition may vary depending on its origin (i.e., laundry, kitchen, or bathroom). For example, kitchen greywater has been shown to have the highest COD, TSS, and turbidity values compared to water from bathrooms or laundry [16,28].

The characterization results indicate that the raw greywater under scrutiny in this study exhibits conventional pollutant loads necessitating efficacious treatment. The relatively elevated values of COD, turbidity, and TSS render it a suitable candidate for the evaluation of the performance of the EC process, particularly with regard to the efficiency of organic matter and solids removal.

### 2.2. Electrode Characteristics

**Figure 1:**
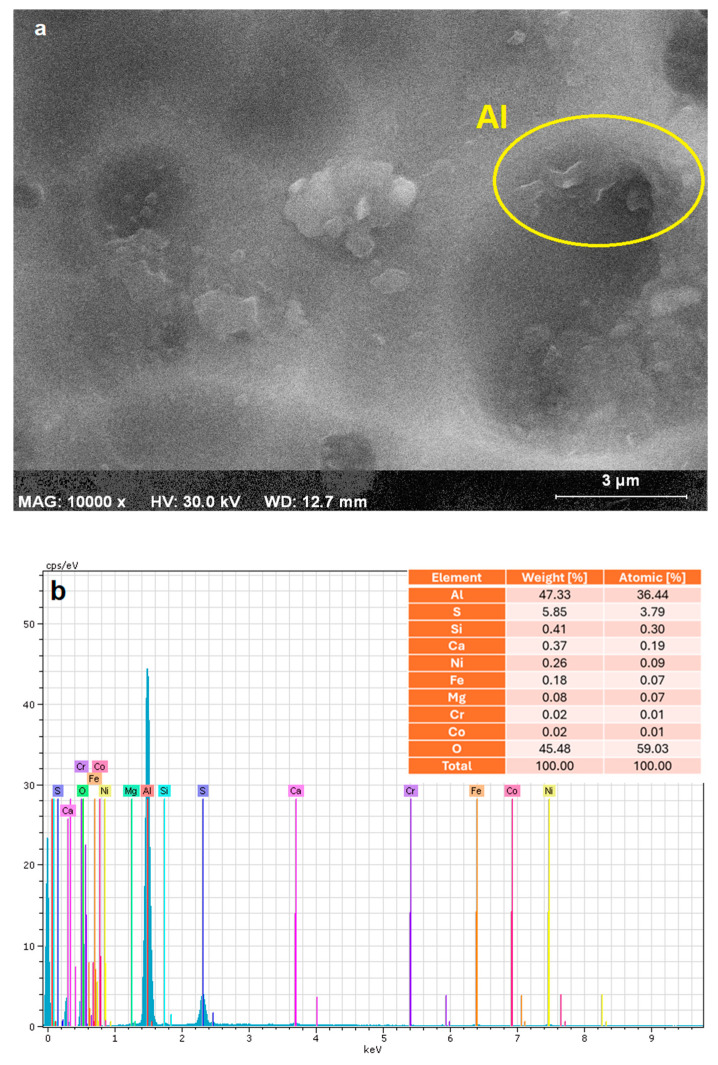
(**a**). SEM image of the Al electrode surface at 10,000× magnification (scale bar: 3 µm), (**b**). EDS spectrum of the Al electrode surface showing elemental composition before EC treatment process.

The electrocoagulation system employed a total of seven aluminum electrodes in this study. To examine the morphological and elemental properties of the electrode material prior to application, Scanning Electron Microscopy (SEM) and Energy-Dispersive X-ray Spectroscopy (EDS) analyses were performed. Figure 1a presents the SEM image of the Al electrode surface before EC treatment process at a magnification of 10,000×. The surface displays a heterogeneous and irregular microstructure, characterized by localized protrusions and depressions. Such surface roughness is beneficial for EC applications, as it enhances the effective electrode surface area and facilitates the formation and detachment of coagulant flocs. The observed texture also suggests the possible presence of surface oxide formations or mild pitting due to prior exposure to electrochemical environments.

The corresponding EDS spectrum (Figure 1b) confirms the elemental composition consistent with Al before EC treatment process. The analysis revealed that Al was the dominant constituent, comprising 47.33 wt% and 36.44 at%, confirming that the precipitates were primarily composed of aluminum hydroxide and oxyhydroxide species, such as Al(OH)_3_ and AlOOH. This is consistent with expectations from the EC mechanism using Al electrodes, where anodic dissolution generates Al^3+^ ions that subsequently hydrolyze and precipitate under near-neutral pH conditions [29].

Additionally, sulfur (S) was present at a notable level (5.85 wt%), potentially originating from sulphate ions in the treated water or co-precipitated basic aluminum sulphates. The detection of trace elements such as Si, Ca, Mg, Fe, Cr, Ni, and Co (collectively <2 wt%) may be attributed to either background water matrix contaminants or minor electrode corrosion, particularly in the case of Fe and Cr, which can leach from Al alloys or process equipment [30]. However, the very low atomic percentages of Fe (0.07%) and Cr (0.01%) suggest minimal contamination from stainless steel or other components, reinforcing the interpretation that the sludge is primarily Al-based.

### 2.3. Effect of Current Density

**Figure 2:**
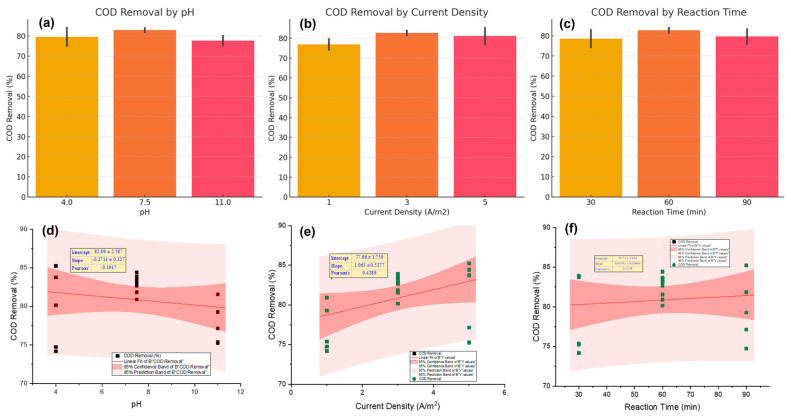
Effect of pH, current density, and reaction time on COD removal: (**a**–**c**) experimental results; (**d**–**f**) regression plots with 95% confidence intervals.

Current density, a critical factor controlling the rate of coagulant generation and gas bubble formation, also exhibited a pronounced effect on treatment performance. Figure 2 (center) illustrates the linear relationship between current density and COD removal efficiency in the EC process. The maximum COD removal (~83%) was recorded at a current density of 3 A/m^2^, suggesting an optimal balance between coagulant production and energy input. At lower current densities (such as 1 A/m^2^), the limited release of metal ions and reduced gas production can hinder the formation of flocs, which in turn lowers the treatment efficiency. On the other hand, while COD removal was generally effective at higher current densities, a slight drop was noted at 5 A/m^2^. This may be due to excessive gas generation causing turbulence in the system, which disrupts floc formation—an observation that aligns with previous studies by Bajpai et al. [2] and Awasthi et al. [9]. Furthermore, higher current densities may also cause passivation of the electrode surface, exerting a negative effect on the release of coagulants and overall system performance. An overall increasing trend can be observed, indicating that higher current densities allow for better removal of organic pollutants. This observation agrees with many recent studies highlighting current density as an important parameter influencing the efficiency of electrochemical treatments [31]. The second panel illustrates a statistically moderate positive correlation between current density and COD removal efficiency, characterized by a slope of 1.063 ± 0.5277 and a Pearson’s correlation coefficient (r) of 0.4289. This relationship indicates that increasing current density facilitates the enhanced generation of coagulant species, thereby improving pollutant destabilization and promoting floc formation [32]. As the applied current increases, the anodic dissolution of iron or chromium electrodes intensifies, leading to increased production of hydroxide precipitates. These hydroxides contribute to COD reduction through adsorption and charge neutralization mechanisms. However, the presence of moderate dispersion around the trendline suggests diminishing returns at higher current values, which is consistent with the results of Reátegui-Romero et al. [33], who emphasized the importance of balancing removal efficiency with energy consumption.

### 2.4. Effect of Reaction Time

Figure 2 (right) shows how reaction time affects the efficiency of COD removal. The results indicate that a 60-min electrolysis duration yielded the highest mean COD removal among the three examined time intervals (30, 60, and 90 min). In other words, the removal of COD improved a lot from 30 to 60 min, but there was only a small drop or a plateau at 90 min. This observation is in accordance with the recent findings indicating the time-dependent nature of pollutant removal EC systems. However, it is well-documented that extending the reaction time beyond a certain operational threshold does not necessarily lead to further improvements in treatment efficiency. For instance, Patel et al. [34] reported that prolonging the reaction time beyond the optimal point can result in diminishing returns due to coagulant saturation and potential restabilization of colloidal particles. In a similar vein, Mahmad et al. [35] emphasized that overly extended electrolysis durations may trigger undesirable side reactions, including the evolution of oxygen and hydrogen gases. These gases have the capacity to interfere with the process of floc agglomeration and even promote the re-dispersion of previously settled solids.

Furthermore, extended treatment durations have been shown to increase energy consumption and accelerate electrode wear, without providing proportional gains in removal efficiency [4]. This was demonstrated in an investigation of greywater treatment using aluminum and stainless steel electrodes. Consequently, ascertaining an optimal reaction time is imperative to ensure an equilibrium between treatment efficacy and operational expense.

In the present study, enhanced removal efficiency was observed at 60 min, likely due to the formation of stable and adequately sized flocs that had sufficient contact time to capture pollutants and undergo effective sedimentation. A slight decrease in performance at 90 min may be attributed to floc disintegration, interference from excessive gas evolution, or electrochemical passivation of the electrode surfaces. These results highlight the need for process optimization using tools like RSM that can establish the exact operational window for which to achieve maximum efficiency at minimum energy input and electrode degradation. The third panel illustrates the influence of electrolysis duration on COD removal efficiency. A slight positive slope (0.0191 ± 0.0386) and a weak correlation coefficient (r = 0.1158) reveal that extended reaction times contribute marginally towards COD removal. This is in line with the fundamental principle that longer treatment allows for greater interaction between pollutants and coagulating species [36]. Nonetheless, the weak correlation observed suggests that beyond a certain point, prolonged reaction time does not substantially increase COD removal, possibly due to the saturation of available active sites or the re-dissolution of flocs. Similar behavior was reported by Patel et al. [34], who found that 60 min was an optimal duration for achieving significant removal without unnecessary energy input.

### 2.5. Effect of pH

The influence of initial pH on COD removal efficiency during EC was found to be significant (Figure 2, left). The highest removal performance was observed at neutral pH (7.5), where the COD removal efficiency reached its peak (~83%). This behavior aligns with the findings of Al-Qodah et al. [37] and Mousazadeh et al. [38], who reported that neutral conditions favor the formation of flocculent metal hydroxides such as Fe(OH)_3_ or Al(OH)_3_, which play a pivotal role in adsorbing and aggregating organic pollutants. At lower pH (4.0), although the metal dissolution rate increases, the predominance of soluble ionic species and reduced floc stability may hinder effective pollutant capture. Conversely, at alkaline pH (11.0), the decline in COD removal can be attributed to the precipitation of large, less reactive hydroxide particles and increased electrostatic repulsion, as also noted by Ansari et al. [4]. The first panel of Figure 2 presents the influence of initial pH on COD removal efficiency. The negative slope (−0.2714 ± 0.327) and the Pearson correlation coefficient (r = −0.1917) suggest a weak and inverse relationship between pH and COD removal. Although EC generally operates efficiently in the slightly acidic to neutral pH range (6–8), the minimal effect observed here aligns with studies indicating that Al electrodes maintain stable performance across a broader pH spectrum [39,40]. The weak correlation suggests that, within the range of pH values examined (4–11), other parameters such as current density or electrolysis time may exert a more significant influence. This observation is consistent with the findings of Mousazadeh et al. [38], who reported a comparable negligible impact of pH on COD removal from domestic effluents when Al electrodes were utilized.

### 2.6. RSM Optimization

The experimental studies were executed in alignment with the design matrix formulated through RSM (BBD), with the removal of COD from greywater documented as the response variable. RSM can be implemented through various experimental designs such as Central Composite Design (CCD), Definitive Screening Design (DSD), and BBD. Among these, the BBD applied in the current study offers distinct advantages for modeling COD removal via electrocoagulation. Unlike CCD, which requires axial points at extreme values, BBD avoids impractical operating conditions and ensures that all design points fall within a moderate and safe operational range. Compared to DSD, which is highly efficient in factor screening but less effective in capturing quadratic interactions, BBD provides a better balance between reduced experimental runs and the ability to model nonlinear and interactive effects [4,12].

Recent studies have reported COD removal efficiencies exceeding 85–90% with RSM–BBD optimization for real greywater and industrial effluents, with improved prediction reliability compared to DSD or CCD approaches [9,38]. In the present study, the use of RSM–BBD yielded a statistically significant second-order model with high predictive performance, confirming its suitability for COD removal optimization. This indicates that RSM–BBD is not only experimentally economical but also capable of accurately describing the complex interactions among pH, current density, and electrolysis time in EC processes. The results from these experimental runs were then fitted to the quadratic model shown in Equation (1).

(1) $$COD\;Removal\left(\%\right)=82.34-0.29A+1.72B+0.12C-3.33AB-0.12AC+0.47BC-2.5A^{2}-0.74B^{2}-1.13C^{2}$$

The quadratic model developed from the BBD (see Equation (1)) provides significant insights into the individual and interactive effects of pH (A), current density (B), and reaction time (C) on COD removal efficiency. The constant term (82.34) represents the default rate of removal under standardized conditions, while the positive coefficient of current density (+1.72) indicates a significant direct effect on COD removal, validating literature highlighting the pivotal role of current in promoting the formation of coagulants and the destabilization of impurities [41]. Conversely, pH has a slightly negative linear effect (−0.29), showing that deviations from the optimal neutral range might work against process efficiency, plausibly due to changes in floc formation kinetics or electrode dissolution behavior [42]. The reaction time has an inconsequential positive effect (+0.12), implying minimal improvements beyond a time threshold, which aligns with findings showing that longer electrolysis may bring diminishing returns [37]. The interaction terms show that the interaction between pH and current density (−3.33) has a pronounced antagonistic impact, highlighting the importance of ensuring balanced settings to avoid counterproductive outcomes. The negative quadratic terms of every variable (A^2^, B^2^, C^2^) confirm the presence of nonlinear effects and further support the occurrence of optimal operating ranges, where treatment efficiency declines beyond these ranges. This model underscores the need for multi-variable optimization for maximal COD removal in EC processes. The experimental matrix in Table 2 reports the 20-run BBD used to investigate the influence of pH (A), current density (B), and reaction time (C) on COD removal efficiency from real greywater via Al-based EC. Within the 20 experimental runs, COD removal varied between 73.09% and 84.13%, illustrating the process sensitivity to the operational parameter variations. The maximum COD removal (84.13%) occurred at low pH (4), high current density (5 A/m^2^), and extended reaction time (90 min), reflecting favorable conditions for electrocoagulant formation and pollutant agglomeration under these conditions. In contrast, the minimum removal (73.09%) was obtained at the same pH but with both current density and electrolysis time set at their minimum levels (1 A/m^2^, 30 min), likely due to an inadequate coagulant dosage and contact time at these levels. The central points (runs with pH 7.5, current density 3 A/m^2^, and reaction time 60 min) were repeated to estimate the experimental error and model reproducibility. These replicates consistently yielded COD removal efficiencies between 81.37% and 82.59%, affirming the reliability of the experimental design and the stability of the EC system under mid-level operating conditions.

These results further emphasize the significance of interactive and quadratic effects between process variables, as previously confirmed by the ANOVA results described in Section 2.8 and surface response analyses. The findings support the conclusion that optimizing operational conditions—particularly current density and treatment time—is critical for maximizing COD removal in real greywater treatment applications.

### 2.7. ANOVA-Based Statistical Evaluation of Experimental Data

ANOVA analysis was conducted by taking pH, current density, and reaction time as the independent variables (Table 3). An analysis of variance (ANOVA) was employed to evaluate the statistical significance and adequacy of the quadratic model developed for COD removal using EC. As presented in Table 3, the model is highly significant, as evidenced by a high F-value of 24.38 and a *p*-value less than 0.0001, indicating that the regression model reliably explains the variation in COD removal.

Among the individual linear terms, current density (B) demonstrated a statistically significant effect (F = 33.40, *p* = 0.0002), confirming its dominant role in driving the EC process, which is consistent with recent studies [9,43]. In contrast, pH (A) and reaction time (C) did not show statistically significant individual effects at the 95% confidence level (*p* = 0.3579 and 0.7026, respectively), suggesting that within the tested range, their independent contributions to COD removal are limited or nonlinear in nature. Importantly, the interaction term AB (pH × current density) was highly significant (F = 100.19, *p* < 0.0001), indicating a strong synergistic or antagonistic interplay between these two variables. This highlights the necessity of jointly optimizing pH and current density, as their combined effect substantially influences the process performance. Other interaction terms (AC and BC) and quadratic terms (B^2^, C^2^) were not statistically significant, although A^2^ was significant (*p* = 0.0013), indicating a nonlinear relationship between pH and COD removal efficiency. The model’s residual mean square error (0.8868) is relatively low, reflecting a good fit between predicted and observed values. Although the lack of fit was found to be statistically significant (F = 7.92, *p* = 0.0002), this may be attributed to the variability inherent in real greywater matrices, which are known to contain fluctuating levels of organic load and surfactants that may not be fully captured by second-order models. Despite this, the model still provides a valuable predictive framework within the tested parameter space and is consistent with similar studies employing Box–Behnken designs for real wastewater treatment applications.

The model was found to be statistically significant with a high F-value of 24.38 and a *p*-value of <0.0001, indicating that the model adequately explains the variation in COD removal within the studied range of parameters. Furthermore, the model fit statistics further reinforce its robustness. The coefficient of determination (R^2^) was 0.96, indicating that 96% of the variance in COD removal was explained by the model. The adjusted R^2^ (0.92) and predicted R^2^ (0.85) values were also high and in reasonable agreement, demonstrating both explanatory power and predictive reliability. The coefficient of variation (CV) was low (1.17%), suggesting high precision, while the Adequate Precision value of 16.58 (well above the threshold of 4.0) indicates an excellent signal-to-noise ratio for navigating the design space.

The ANOVA results validate the model’s overall robustness, particularly emphasizing the significant influence of current density and its interaction with pH. These findings underscore the need for multidimensional optimization in EC processes targeting real greywater treatment.

**Figure 3:**
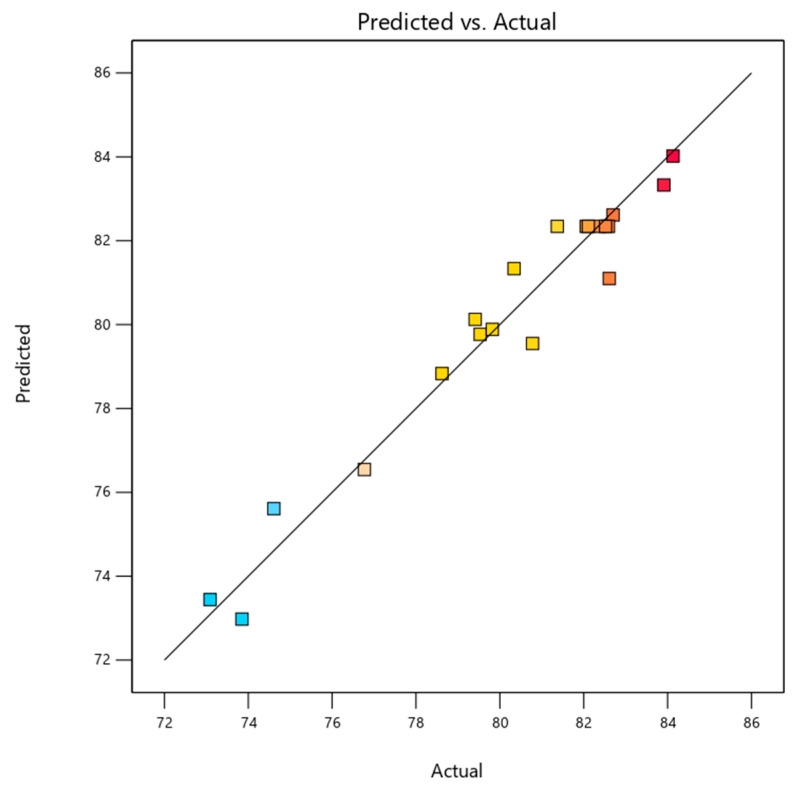
Comparison between the experimental and model-predicted COD removal efficiencies using the BBD.

The scatter plot as shown in Figure 3 presents the predicted versus actual COD removal efficiencies obtained from the EC experiments designed using the BBD. The data points, represented as colored squares, illustrate how closely the model predictions align with experimental observations across varying combinations of pH, current density, and reaction time. The strong linear distribution of the data along the 45-degree diagonal (line of equality) suggests excellent agreement between the experimental results and the model-predicted values, a hypothesis that is further supported by the minimal deviation of most points from the line. This finding suggests that the quadratic regression model developed for COD removal demonstrates a high degree of predictive accuracy and reliability. The color gradient, ranging from blue (lower COD removal) to red (higher COD removal), further illustrates the distribution of removal efficiencies across the experimental domain. The best efficiency was 84.13%, and the worst was 73.09%. The model’s accuracy aligns with the findings documented in the existing literature. For example, Bajpai et al. [2] and Pacheco et al. [44] have shown that BBD-based RSM models can accurately predict how well contaminants will be removed in EC processes. These results show that the statistical design used in this study works well for capturing nonlinear interactions between operational parameters and setting up a framework for optimizing the process of treating real greywater.

**Figure 4:**
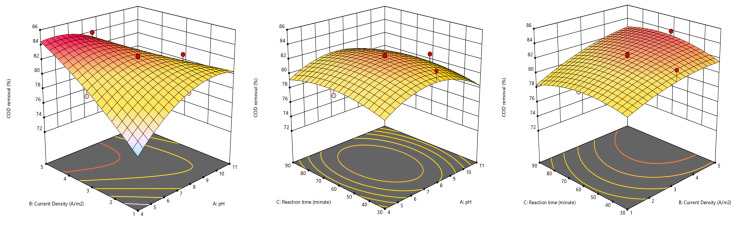
3D RSM (BBD) plots showing the interactive effects of EC process parameters during greywater treatment on COD removal efficiency.

The 3D response surface plots (Figure 4) provide a visual summary of how pH, current density, and reaction time work together to influence COD removal during the EC treatment of greywater. Among the tested parameters, the most significant interaction was observed between pH and current density. Higher removal efficiencies were generally achieved when the pH was in the range of 4 to 6 and the current density was kept at moderate levels. This can be explained by the increased release of metal ions and the optimal formation of coagulant species under these conditions, which promote the effective destabilization and aggregation of organic matter [25,36]. In contrast, at higher pH values, flocs tend to become less soluble, which reduces treatment effectiveness. The interaction between pH and reaction time had a less noticeable effect. This suggests that extending the reaction time beyond a certain point—such as beyond 60 min—does not provide significant additional benefits, especially when the pH conditions are not ideal. This observation aligns with previous findings indicating that prolonged treatment may not enhance removal significantly once the system reaches coagulant saturation or when floc stability is compromised [4]. The response surface for current density and reaction time revealed a clear synergistic effect, where increasing both parameters improved COD removal up to an optimal zone. However, excessively high current densities combined with long reaction times may lead to unnecessary energy consumption and possible re-dissolution of formed flocs [3]. All these results emphasize that while each parameter individually influences performance, their interactions play a critical role in optimizing the EC process for greywater treatment. The fitted quadratic model effectively captures these nonlinear relationships, supporting its use as a reliable tool for process optimization.

### 2.8. Kinetic Modelling of COD Removal

**Figure 5:**
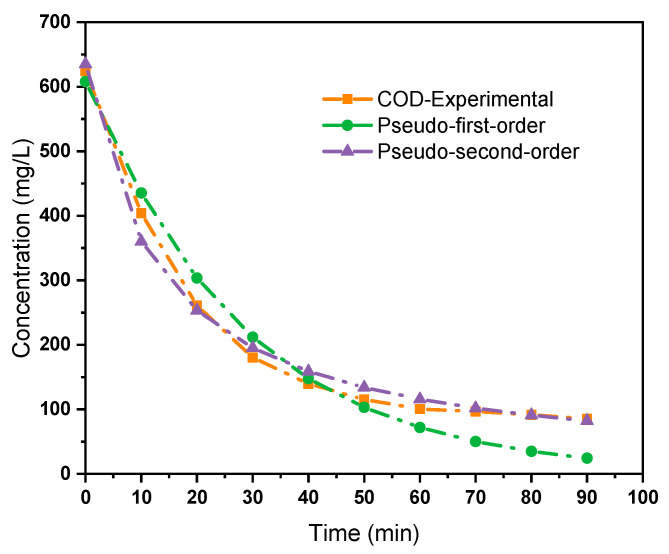
Comparison of experimental and model-predicted COD concentrations over time using pseudo-first order and pseudo-second-order kinetic models during the EC treatment of greywater.

The temporal evolution of COD concentration was evaluated using pseudo-first-order and pseudo-second-order kinetic models to elucidate the reaction dynamics of the EC process. As shown in Figure 5, both models demonstrated acceptable agreement with the experimental data; however, the pseudo-second-order model provided a better fit across the entire treatment duration. This suggests that the COD removal process may be more accurately described by a chemisorption mechanism, where the adsorption capacity is related to the square of the available active sites on the flocs generated during EC. The initial COD concentration of 624 mg/L decreased to 85.24 mg/L within 90 min under optimal operational conditions (pH 7.5, current density 3 A/m^2^), corresponding to a removal efficiency of approximately 86%. The pseudo-second-order model yielded a more consistent prediction pattern, particularly in the latter stages of treatment, where slower removal kinetics are typically observed due to the depletion of readily coagulated organic matter.

These findings are in agreement with recent studies on EC using aluminum or stainless steel electrodes, which also reported superior performance of the pseudo-second-order kinetic model [3,9]. The better correlation of the second-order model with experimental data implies that the COD removal process in EC systems is governed not only by mass transfer but also by the availability and reactivity of coagulant species formed in situ. Consequently, kinetic modelling confirms the suitability of EC for treating high-strength greywater, especially when operational parameters are optimized for sustained floc formation and pollutant capture.

### 2.9. Sludge Characteristics

The physicochemical and structural characteristics of the sludge formed via EC using Al electrodes were analyzed through SEM, XRD, and FTIR techniques. These complementary methods enabled an in-depth understanding of the morphology, crystallinity, and functional group composition of the electrochemically precipitated solids.

**Figure 6:**
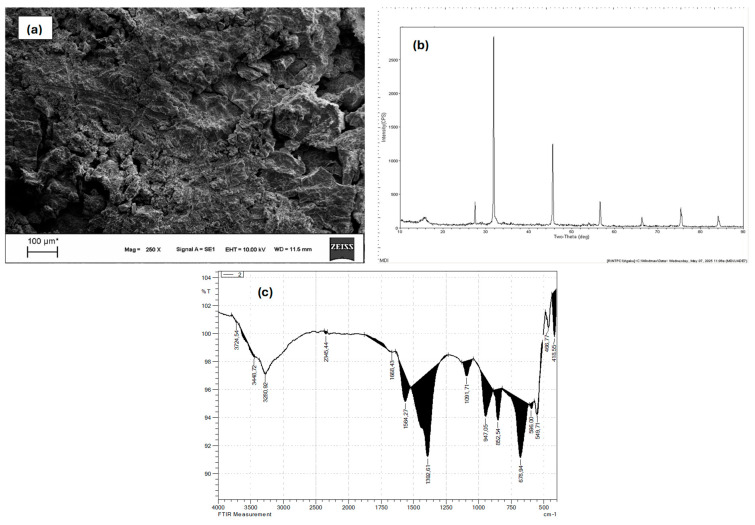
Characterization of sludge generated after the EC process using Al electrodes: (**a**) SEM image, (**b**) XRD spectra, and (**c**) FTIR analysis.

As depicted in Figure 6a, the SEM micrograph illustrates a dense, granular surface texture, with flaky and amorphous aggregate structures. The flocs exhibit a highly irregular morphology, typical of aluminum hydroxide-based coagulation products [34]. The observed layered plate-like regions are characteristic of Al(OH)_3_ precipitation, which acts as a sweep floc during the coagulation process [35]. These flocs exhibit high surface area and interstitial voids, favoring the entrapment of suspended solids and dissolved organics. Such morphological complexity enhances the sludge’s adsorption capacity and promotes rapid settling. The XRD pattern in Figure 6b confirms the presence of poorly crystalline and semi-amorphous phases, which is a hallmark of aluminum hydroxide sludge. The dominant diffraction peaks near 2θ ≈ 18.4°, 39.3°, and 66.5° correspond to bayerite (α-Al(OH)_3_) and boehmite (γ-AlOOH), both of which are common transformation products of anodically dissolved Al under near-neutral pH conditions. The relative broadness of the peaks and the absence of sharp signals indicate a low degree of crystallinity, consistent with rapid precipitation and short aging time of Al-based flocs. The FTIR spectrum shown in Figure 6c reveals several key vibrational bands indicative of hydroxyl and metal-oxygen bonding. The broad absorption at 3428 cm^−1^ is attributed to O–H stretching vibrations from hydroxyl groups and adsorbed water, commonly present in hydrated Al species [44]. The band at 1638 cm^−1^ corresponds to H–O–H bending, indicating structural or interlayer water. Notably, the bands observed between 600–400 cm^−1^, especially at 547 and 464 cm^−1^, are assigned to Al–O and Al–OH bending vibrations, which are typical for aluminum oxyhydroxide species such as boehmite and gibbsite. Minor signals at 1382 cm^−1^ and 1091 cm^−1^ may also reflect the presence of carbonate or nitrate residues entrapped within the floc matrix [45].

The SEM, XRD, and FTIR analyses collectively confirm that the sludge consists primarily of amorphous to poorly crystalline aluminum hydroxides and oxyhydroxides, such as Al(OH)_3_, AlOOH, and trace alumina-like phases. These findings align with literature reporting that Al-based EC yields voluminous, gel-like flocs with high hydration levels and low crystallinity. The morphology and bonding characteristics observed are critical to understanding sludge behavior in terms of dewatering, filterability, and metal residuals in treated water [4,41]. Moreover, these results suggest that pollutant removal in this study primarily occurred through physical adsorption, charge neutralization, and sweep flocculation via electrochemically generated Al(OH)_3_ and AlOOH species, rather than chemical degradation.

### 2.10. Greywater Reuse Potential and Compliance with International Standards

The reuse of treated greywater represents a key strategy for sustainable water management, reducing pressure on freshwater resources and minimizing wastewater discharge. In this study, the optimized aluminum-based EC process substantially improved the physicochemical quality of real greywater collected from kitchen sink and laundry sources. The treatment achieved high pollutant removal efficiencies, particularly in terms of COD, TSS, and turbidity, while maintaining pH within acceptable ranges for reuse applications.

To assess the potential for non-potable reuse, the raw and treated greywater parameters were compared with selected international guidelines from WHO, USEPA, and EU 2020/741 (Table 1).

The treated greywater met or exceeded all tested thresholds for non-potable reuse across WHO, USEPA, and EU guidelines. Particularly notable is the compliance with the strict USEPA limit for turbidity (≤2 NTU) and COD (≤50 mg/L), which are critical parameters for urban reuse in applications involving potential human contact. These findings indicate that the electrocoagulation process not only yields treated greywater that meets the quality requirements for agricultural irrigation but also satisfies the more stringent standards for unrestricted urban reuse, thereby contributing meaningfully to resource recovery efforts and advancing circular economic principles.

## 3. Materials and Methods

**Figure 7:**
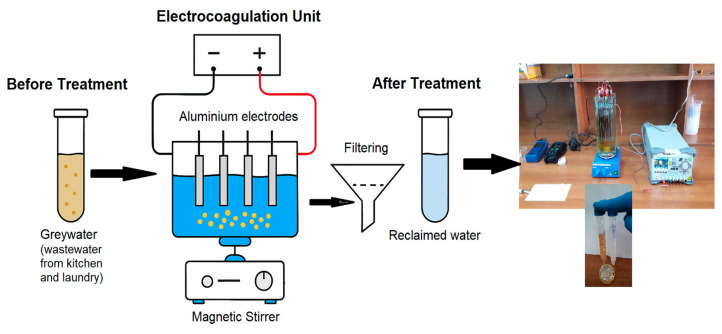
Schematic representation of the experimental system and the conceptual framework employed in this study.

This section consists of three subsections: Greywater Sampling and Characterization, Experimental Setup and Procedure, and RSM. Figure 7 provides a visual representation of the system configuration and the underlying conceptual framework of the research.

### 3.1. Greywater Sampling and Characterization

In this study, real greywater was used to assess the EC process under realistic domestic conditions. Greywater samples were collected from three separate residential buildings located in Sanliurfa, Türkiye over five consecutive days. Wastewater was drawn from two primary sources: kitchen sinks and laundry discharge points, during peak usage hours (typically 7:00–10:00 a.m. and 6:00–9:00 p.m.) to capture typical pollutant fluctuations. A total volume of approximately 60 L of raw greywater was obtained. The individual samples were homogenized and composited into a single bulk representative sample to minimize variability between households and ensure consistency in experimental treatment and analysis. Samples were collected in high-density polypropylene containers, transported immediately to the laboratory, and stored at 4 °C to preserve their physicochemical integrity prior to treatment.

Initial greywater quality was characterized by measuring pH, COD, EC, turbidity, and TSS. pH and EC were measured using a Hach HQ40D multi-parameter meter (Hach Company, Loveland, CO, USA). COD was determined using a WTW CR 4200 COD Thermoreactor (Xylem Analytics, Weilheim, Germany) in accordance with American Public Health Association [46] standard dichromate digestion method. Turbidity measurements were performed with an OHAUS AP30TURH turbidity meter (OHAUS Corporation, Parsippany, NJ, USA), while TSS was determined gravimetrically after filtration through 0.45 µm glass fiber filters, followed by drying at 105 °C using a Memmert UF55 oven (Memmert GmbH + Co. KG, Schwabach, Germany).

### 3.2. Experimental Setup and Procedure

Electrocoagulation experiments were conducted in a 1.5 L capacity glass batch reactor containing 1.0 L of greywater. The reactor was equipped with seven rectangular Al electrodes (dimensions 10 cm × 3 cm × 0.1 cm) arranged in a monopolar configuration with an inter-electrode spacing of 1.0 cm. Electrodes were connected to a Rigol DP832 DC power supply (Rigol Technologies, Beijing, China), and continuous mixing was provided using an IKA RW 20 digital overhead stirrer (IKA-Werke GmbH & Co. KG, Staufen, Germany) at 300 rpm to ensure homogeneous treatment conditions.

Each experimental run was performed for reaction times ranging from 0 to 90 min. Prior to each run, electrodes were cleaned with 1 M HCl to remove oxide layers and maintain surface activity. Following EC, samples were filtered and analyzed for COD to evaluate treatment performance.

The COD removal efficiency (%) was calculated using the following Equation (2):

(2) $$COD\;Removal\;Efficiency\left(\%\right)=\left(\frac{C_{i}-C_{f}}{C_{i}}\right)\times 100$$

where C_i_ and C_f_ are the initial and final COD concentrations (mg/L), respectively.

The sludge samples were processed by collecting the precipitates post-treatment, followed by vacuum filtration, and subsequently oven-dried at 60 °C for 24 h to ensure complete moisture removal prior to characterization analyses.

### 3.3. Response Surface Methodology

Process optimization and modelling were conducted using RSM, employing the BBD to investigate the effects and interactions of key operational parameters on COD removal efficiency. Three independent variables were selected: initial pH (A), current density (B), and electrolysis time (C). A total of 20 experimental runs were generated by the BBD matrix, including replication at the center point to estimate experimental error.

The design was based on the following second-order polynomial Equation (3):

(3) $$Y=\beta 0+\sum \beta iXi+\sum \beta iiXi^{2}+\sum \beta ijXiXj$$

where Y is the predicted response (COD removal, %), β0 is the intercept, βi, βii, and βij are the linear, quadratic, and interaction coefficients, respectively, and Xi and Xj are the coded independent variables.

Experimental design, regression analysis, and surface plots were generated using Design-Expert^®^ software (Version 13, Stat-Ease Inc., Minneapolis, MN, USA). The optimal operating conditions for maximum COD removal were identified based on numerical optimization and desirability functions.

### 3.4. Kinetic Study

To evaluate the mechanism and rate-controlling steps of COD removal during the EC process, kinetic modelling was performed using pseudo-first-order and pseudo-second-order models. The pseudo-first-order model assumes that the rate of removal is directly proportional to the difference between the current and equilibrium COD concentrations. Its linearized form is given by Equation (4):

(4) $$q_{t}=q_{e}\left(1-e^{\left(-k_{1}t\right)}\right)$$

where q_t_ is the amount of COD removed at time t (mg/L), q_e_ is the amount of COD removed at equilibrium (mg/L), k_1_ is the first-order rate constant (1/min), t is the reaction time (min).

The pseudo-second-order model assumes that the rate-limiting step may involve chemisorption, and is described by the following linearized Equation (5):

(5) $$q_{t}=\left(k_{2}{q_{e}}^{2}t\right)/\left(1+k_{2}q_{e}t\right)$$

where k_2_ is the second-order rate constant (L/mg min), and all the other terms are as defined above.

## 4. Conclusions

This study demonstrates the applicability and optimization of Al-based EC for the effective treatment of real greywater. Using RSM via a Box–Behnken Design, optimal operating conditions were identified, resulting in a maximum COD removal efficiency of 86.34%. Among the tested parameters, current density emerged as the most significant factor, with a strong interaction with pH also playing a crucial role in process efficiency. The developed quadratic model showed high statistical reliability and predictive capability (R^2^ = 0.96), confirming the robustness of the optimization approach. Sludge analyses using SEM, XRD, and FTIR revealed the predominance of amorphous and poorly crystalline aluminum hydroxide species, which are known for their excellent flocculating and adsorptive properties. These findings confirm the physical adsorption and sweep flocculation mechanisms that dominate pollutant removal in Al-based EC systems. While this study confirms that EC is a viable and sustainable treatment technology for domestic greywater, especially for water reuse in irrigation and other non-potable applications, further investigations are warranted to fully assess its large-scale applicability. Future work should consider life-cycle assessments (LCA) to quantify the environmental footprint, operating cost evaluations for economic feasibility, and microbial risk profiling to ensure compliance with health standards for reuse. Incorporating these aspects will enable a more holistic evaluation of EC as a decentralized greywater treatment solution in real-world scenarios.

## Figures and Tables

**Table 1:** Physicochemical characteristics of real greywater samples before and after electrocoagulation treatment, along with international reuse standards.

Parameter	Unit	Raw Greywater	Treated Greywater	WHO [20]	USEPA [21]	EU 2020/741 [22]
pH	–	8.1	7.5	6.0–9.0	6.5–8.5	6.0–9.0
COD	mg/L	624	85.24	—	≤50	≤125
Turbidity	NTU	74	1.8	—	≤2	≤5
TSS	mg/L	286	4.5	≤30	≤5	≤35
EC	µg/L	1350	865	—	—	—

**Table 2:** Experimental design matrix based on BBD showing the effects of various parameter levels on COD removal efficiency (%).

	Factor 1	Factor 2	Factor 3	Response 1
Run	A: pH	B: Current Density	C: Reaction Time	COD Removal
		A/m^2^	min	%
1	11	3	60	80.78
2	7.5	3	60	82.59
3	11	5	30	74.61
4	7.5	3	90	80.34
5	7.5	3	60	82.06
6	7.5	1	60	79.82
7	11	5	90	76.77
8	7.5	3	60	82.37
9	4	1	90	73.85
10	7.5	5	60	83.91
11	7.5	3	30	82.61
12	4	3	60	79.41
13	7.5	3	60	82.11
14	7.5	3	60	82.52
15	11	1	90	78.62
16	4	1	30	73.09
17	11	1	30	79.53
18	4	5	30	82.7
19	7.5	3	60	81.37
20	4	5	90	84.13

**Table 3:** ANOVA results and model fit statistics for the quadratic model describing the effects of pH, current density, and reaction time on COD removal efficiency.

**ANOVA Results**							
**Source**	**Sum of Squares**	**df**	**Mean Square**	**F-Value**	***p*-Value**		
Model	194.60	9	21.62	24.38	<0.0001	significant	
A-pH	0.8237	1	0.8237	0.9288	0.3579		
B-Current Density	29.62	1	29.62	33.40	0.0002		
C-Reaction time	0.1369	1	0.1369	0.1544	0.7026		
AB	88.84	1	88.84	100.19	<0.0001		
AC	0.1105	1	0.1105	0.1245	0.7315		
BC	1.75	1	1.75	1.97	0.1906		
A^2^	17.26	1	17.26	19.46	0.0013		
B^2^	1.49	1	1.49	1.68	0.2246		
C^2^	3.48	1	3.48	3.92	0.0757		
Residual	8.87	10	0.8868				
Lack of Fit	7.87	5	1.57	7.92	0.0002	significant	
Pure Error	0.9946	5	0.1989				
Cor Total	203.47	19					
**Model Fit Statistics**							
**Targeted Compounds**	**Std. Dev.**	**Mean**	**C.V. %**	**R^2^**	**Adjusted R^2^**	**Predicted R^2^**	**Adeq Precision**
COD	0.94	80.16	1.17	0.96	0.92	0.85	16.58

## Data Availability

The original contributions presented in this study are included in this article; further inquiries can be directed to the corresponding author.
